# Relationship between Water Soluble Carbohydrate Content, Aphid Endosymbionts and Clonal Performance of *Sitobion avenae* on Cocksfoot Cultivars

**DOI:** 10.1371/journal.pone.0054327

**Published:** 2013-01-14

**Authors:** Hussein Alkhedir, Petr Karlovsky, Stefan Vidal

**Affiliations:** 1 Department of Crop Sciences, Agricultural Entomology, Georg-August University, Goettingen, Germany; 2 Department of Crop Sciences, Molecular Phytopathology and Mycotoxin Research, Georg-August University Goettingen, Goettingen, Germany; Ben-Gurion University of the Negev, Israel

## Abstract

Aphids feed on plant phloem sap, rich in sugars but poor in essential amino acids. However, sugars cause osmotic regulation problems for aphids, which they overcome by hydrolysing the sugars in their gut and polymerising the hydrolysis products into oligosaccharides, excreted with honeydew. Aphids harbour primary bacterial endosymbionts, which supply them with essential amino acids necessary for survival. They also harbour secondary (facultative) endosymbionts (sfS), some of which have a positive impact on life history traits, although it is not yet known whether they also play a role in providing effective tolerance to differing levels of water soluble carbohydrates (WSCs). We investigated the relationship between WSC content of cocksfoot cultivars and performance of clones of the English grain aphid *Sitobion avenae* F. We evaluated how clone genotype and their sfS modulate performance on these different cultivars. We therefore examined the performance of genetically defined clones of *S. avenae*, collected from different host plants, harbouring different sfS. The performance was tested on 10 *Dactylis glomerata* L. cultivars with varying WSC content. *D. glomerata* is known as a wild host plant for *S. avenae* and is also commercially planted. We found that high WSCs levels are responsible for the resistance of *D. glomerata* cultivars to specific *S. avenae* clones. The minimum level of WSCs conferring resistance to *D. glomerata* cultivars was 1.7% dw. Cultivars with a WSC content of 2.2% or higher were resistant to *S. avenae* and did not allow reproduction. Our results further indicate that sfS modulate to some extend host plant cultivar adaptation in *S. avenae*. This is the first study revealing the importance of WSCs for aphid performance. Cocksfoot cultivars with a high content of WSCs might be therefore considered for aphid control or used for resistance breeding in this and other grass species, including cereals.

## Introduction

Several sucking herbivores, such as aphids and whiteflies, have adapted to the highly specialized phloem sieve elements of plants as their feeding sites. Despite this adaptation, the composition of the ingested phloem sap still does not match the nutritional requirements of these insects in terms of quantity and profile of essential amino acids and the concentration of water soluble carbohydrates. However, an obligatory symbiotic bacterium, *Buchnera aphidicola,* housed in specific bacteriocytes, is functional in synthesizing essential amino acids *de novo* [1] and these amino acids can be used by host aphids. In order to take up sufficient amounts of amino acids from plants, the aphids ingest large quantities of phloem sap. Because the concentration of water-soluble carbohydrates (WSC) in the phloem sap may exceed 50% (w/v), depending on the environmental conditions [2], [3], plant species, and developmental age [4], respectively, the aphids need to secrete these compounds as honey dew to maintain osmotic neutrality. The major component of WSC in plants is sucrose [5]. The osmotic pressure value of the phloem sap often exceeds that of the insect body fluids by a factor of up to 5 [6]. Without protective mechanisms, the osmotic pressure exerted by soluble carbohydrates on the aphid digestive system would cause loss of water from their body fluids, particularly the haemolymph, leading to fatal dehydration. Therefore, aphids adapt to feeding on large quantities of phloem sap by a two-step conversion of sucrose to products of low osmotic activity. In the first step, sucrose is hydrolyzed into glucose and fructose [7], [8], [9]. The invertase, responsible for this hydrolysis in the gut has been characterized for *Acyrthosiphon pisum* [10]. The products of the hydrolysis are then polymerized into oligosaccharides and excreted with honeydew, whose osmotic pressure is comparable to the aphid’s own haemolymph [5], [11], [12]. A proportion of the hexoses generated from sucrose hydrolysis is reabsorbed by the gut, providing aphids with energy for respiration and carbon intermediates for use in the anabolic pathways [7], [8], [9]. The combined rates of polymerization and reabsorption of sucrose hydrolysis products should substantially exceed the invertase reaction, to prevent an increase in the osmotic pressure of the aphid’s gut contents.

Apart from the identification of invertase as an aphid enzyme [10], it is not known whether the primary bacterial endosymbionts contribute to the conversion of WSC by aphids. Wilkinson et al. [12] showed that *B. aphidicola* is not involved in the hydrolysis of sugar. Price et al. [10] also showed that this primary symbiont is not responsible for sucrose hydrolysis in *Acyrthosiphon pisum.* However, it remains to be analysed whether this endosymbiont is involved in the polymerization of the hydrolysis products.

In addition to obligatory primary endosymbionts, aphids harbour additional bacterial endosymbionts, which are not essential for survival [13], but contribute to their performance and possibly host plant specialization [14], [15], [16]. These so-called secondary endosymbionts are capable of enhancing the aphid’s tolerance to suboptimal temperatures [17] and may also improve its resistance to parasitoids [18] and entomopathogenic fungi [18], [19].

Artificial diets have been a powerful tool in studies of the nutritional requirements of aphids. To date, artificial diet studies have demonstrated that aphids do not survive on dietary sucrose concentrations greater than 1 M or 34% w/v [13]. Circumstantial evidence indicates that the presence of bacterial endosymbionts affects the performance of the pea aphid, *Acyrthosiphon pisum,* when fed a high sucrose diet [13]. As the concentration of sucrose in the plants’ phloem sap is species-specific, this finding indicates that bacterial endosymbionts may affect host plant specialization in aphids.

Plant breeders traditionally selected cultivars with high WSC content, which is regarded as a proxy for drought and salt tolerance in wheat [20] and resistance to brown strip disease in cocksfoot [21]. In rye grass, the WSC concentration is regarded as an indicator of silage quality and a contributor to milk production [22], [23], [24]. Apart from being a weed in wheat fields, *D. glomerata* has been cultivated in Europe, Japan, and elsewhere as a forage grass. Remarkably, the specific role of WSC in the resistance of plants to aphids has not yet been experimentally investigated.

The English grain aphid *Sitobion avenae* (F.) is an important pest of cereals, especially in temperate climates of both the Northern and Southern Hemispheres [25], [26], [27], [28]. *S. avenae* is present throughout the year on grasses of the Gramineae family, including cereals. It is regarded as autoecious, with all life stages occurring on the same host plant species. Cocksfoot (*Dactylis glomerata* L.) is a common weed found in and around cereal fields and a known host of *S. avenae.* Molecular marker analysis, the host plants the aphids have been collected from, and the performance of *S. avenae* clones on the *D. glomerata* cultivar (cv) ‘Prairial’ indicate the existence of specialized *S. avenae* clones onthis grass species [29], [30].

The aim of this study was to examine (i) whether the genotype of *S. avenae* and the host plant the clones have been collected from in the field influence their performance on cocksfoot cultivars, (ii) whether different levels of WSC content relate to the performance of *S. avenae* clones on these cocksfoot cultivars, and (iii) whether secondary endosymbionts are functional in explaining the performance of *S. avenae* on these cocksfoot cultivars varying in WSC content.

## Results

### Impact of Host Origin on Performance and Specialisation

**Figure 1:**
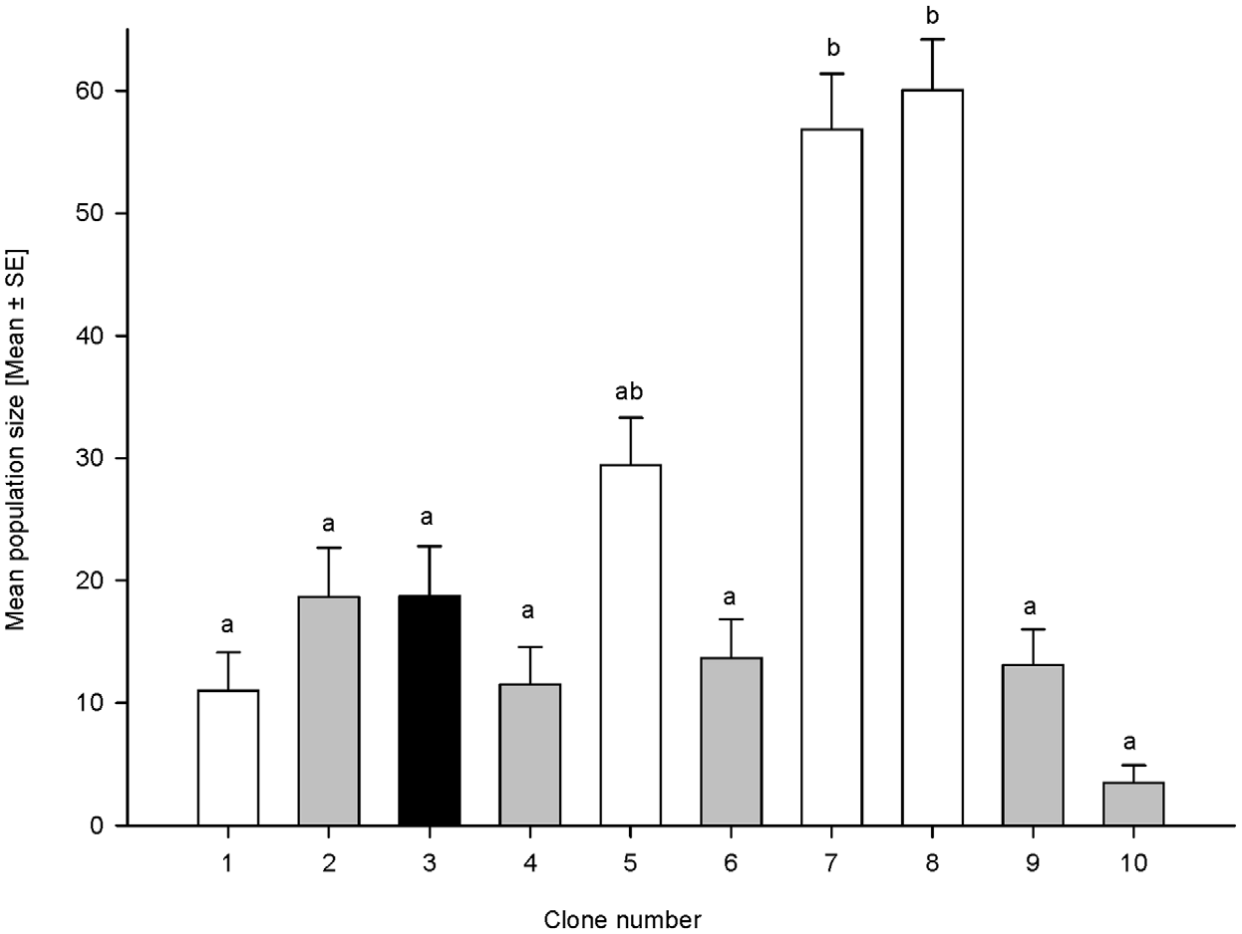
Population (Mean ± SE) of *Sitobion avenae* on 10 cocksfoot cultivars. White bars: clones collected from wheat; black bar: clone collected from *Bromus sp*.; grey bars: clones collected from *Dactylis glomerata*.

We found no relationship between the original plant species, the aphids have been collected from and the performance on cocksfoot cultivars for any of the clones studied. Clones of different genotypes collected from wheat (clones 1, 5, 7 and 8) showed good performance on cocksfoot, while two of the clones (7 and 8) performed betterr on the same host plant (Figure 1). No significant difference (F_1,187_ = 0.001, P>0.05) was observed between the performance of clone 3, which was collected from *Bromus* and clone 2, which was collected from cocksfoot and related to the genotype of clone 1. Both of these clones performed better than the other clones that were collected from cocksfoot (clones 4, 6, 9, and 10; Figure 1).

### Performance of Selected *S. avenae* Clones on Cocksfoot Cultivars

**Figure 2:**
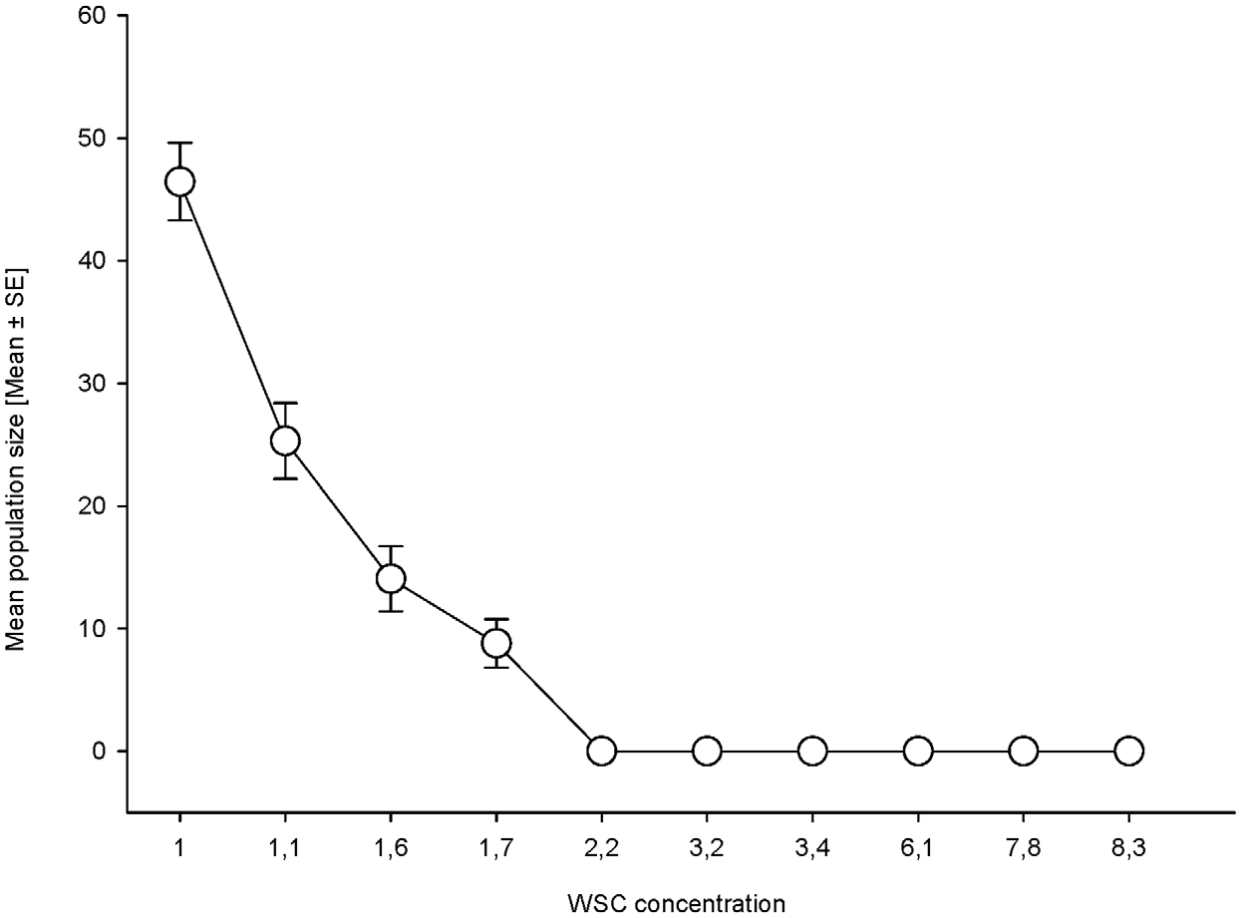
Relationship between mean population size (pooled for the 10 clones tested) and concentration of WSC. Labels on the X axis represent cocksfoot cultivars differing in their WSC content (see Table 2).

**Figure 3:**
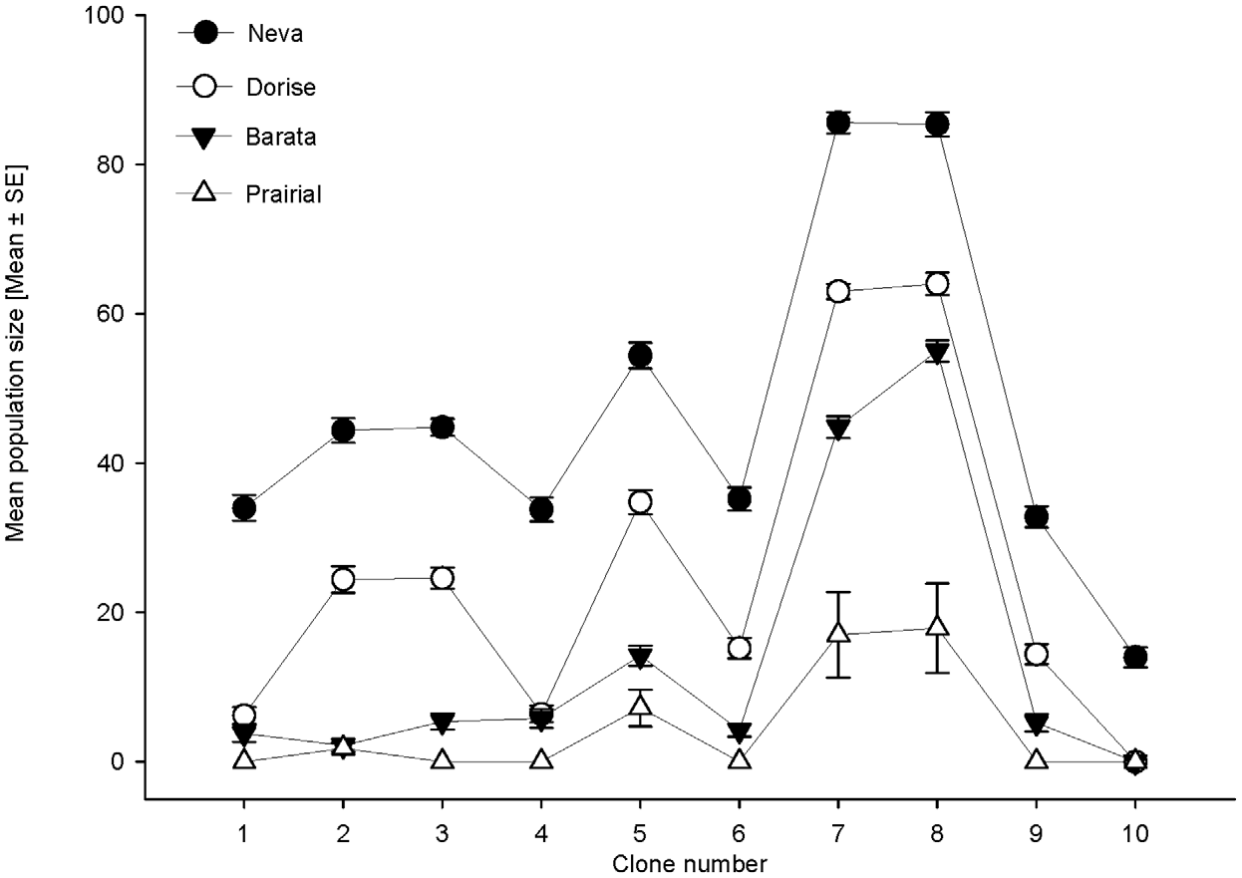
Mean population size of 10 clones *S. avenae* on 4 cocksfoot cultivars. Labels on the X axis refer to the number of the aphid clone tested.

WSC content of the cocksfoot cultivars used in this study varied from 1% to 8.3%. *S. avenae* clones were unable to colonize *D. glomerata* cultivars with a WSC content exceeding 2.2% (Figure 2). None of the specimens that were transferred to these cultivars survived for more than 2 weeks. On cocksfoot cultivars with less than 1.7% WSC, all clones of *S. avenae* survived for the entire duration of the experiment (Figure 3). On cultivar Prairial (containing 1.7% WSC), only clones 5, 7, and 8, all collected from wheat in the field, survived for more than 2 weeks (Figure 3).

The performance of aphid clones from different host plants, harbouring different bacterial endosymbionts (Table 1), significantly varied on the cocksfoot cultivars tested (F_3,187_ = 444.657, P<0.001). Some clones exhibited good performance in terms of offspring production on all cultivars, such as clones 5, 7, and 8, respectively, while other clones, such as clone 10, showed high mortality on all tested cultivars (Figure 1). When the specimens of clones 5, 7, and 8, surviving on cocksfoot cultivars for two weeks, were transferred to wheat plants for 2 weeks and then subsequently transferred to Prairial for 1 month, only clones 7 and 8 were able to survive and produce offspring.

**Table 1:** Collection sites, genotype and secondary symbionts of *Sitobion avenae* clones tested on 10 cocksfoot cultivars.

Clone ID	Genotype^2^	Collectionsite	Host plant	Secondary symbionts
1	I	Goettingen**^1^**	Wheat	*Regiella insecticola*
2	I	Kassel**^2^**	Cocksfoot	*Regiella insecticola*
3	I	Kassel	Bromus sp.	*Regiella insecticola*
4	L	Goettingen	Cocksfoot	*Hamiltonella defensa*
5	L	Giessen	Wheat	*Hamiltonella defensa*
6	L	Kassel	Cocksfoot	No secondary symbiont
7	A	Giessen^3^	Wheat	*Regiella insecticola*
8	V	Giessen	Wheat	*Regiella insecticola*
9	R1	Kassel	Cocksfoot	*Hamiltonella defensa*
10	R2	Kassel	Cocksfoot	*Hamiltonella defensa*

Geographical position of the collection sites: ^1^Goettingen 51°31N/9° 55E, ^2^Kassel 51°19 N/9°29E, and ^3^Giessen 50°35N/9°29E.

On cocksfoot cultivars containing 1.7% or lower levels of WSC, genotype (F_5,187_ = 62.940, P<0.001) and secondary endosymbionts (F_1,187_ = 5.572, P<0.05) significantly affected clonal performance. The presence of the endosymbiont *Hamiltonella defensa* had no significant influence on the performance of clones 4, 9, and 10, while *H. defensa* significantly enhanced the performance of clone 5 on the tested cultivars, even though this clone was unable to survive and produce offspring on the cocksfoot cultivar Prairial. Clones 2, 3, 7, and 8, respectively, hosting *Regiella insecticola*, exhibited significantly better performance on cocksfoot cultivars compared to the clones hosting *Hamiltonella defensa*. However, only clones 7 and 8 were able to survive and produce offspring on Prairial (see above).

The host plant origin of the genotypes did not affect the clonal performance on cocksfoot (F_1,187_ = 0.001, P>0.05).

### Performance of Different Clones on Cultivar Amba

On ocksfoot cultivar Amba, all except a few nymphs of the 65 clones tested died within 5 to 10 days. These surviving few nymphs, which became adults, remained alive only up to 30 days without producing offspring, unambiguously showing that this cultivar is not a suitable host plant for *S. avenae* clones.

### Performance of Clone 5 on Wheat When Previously Feeding on Cultivar Amba

**Figure 4:**
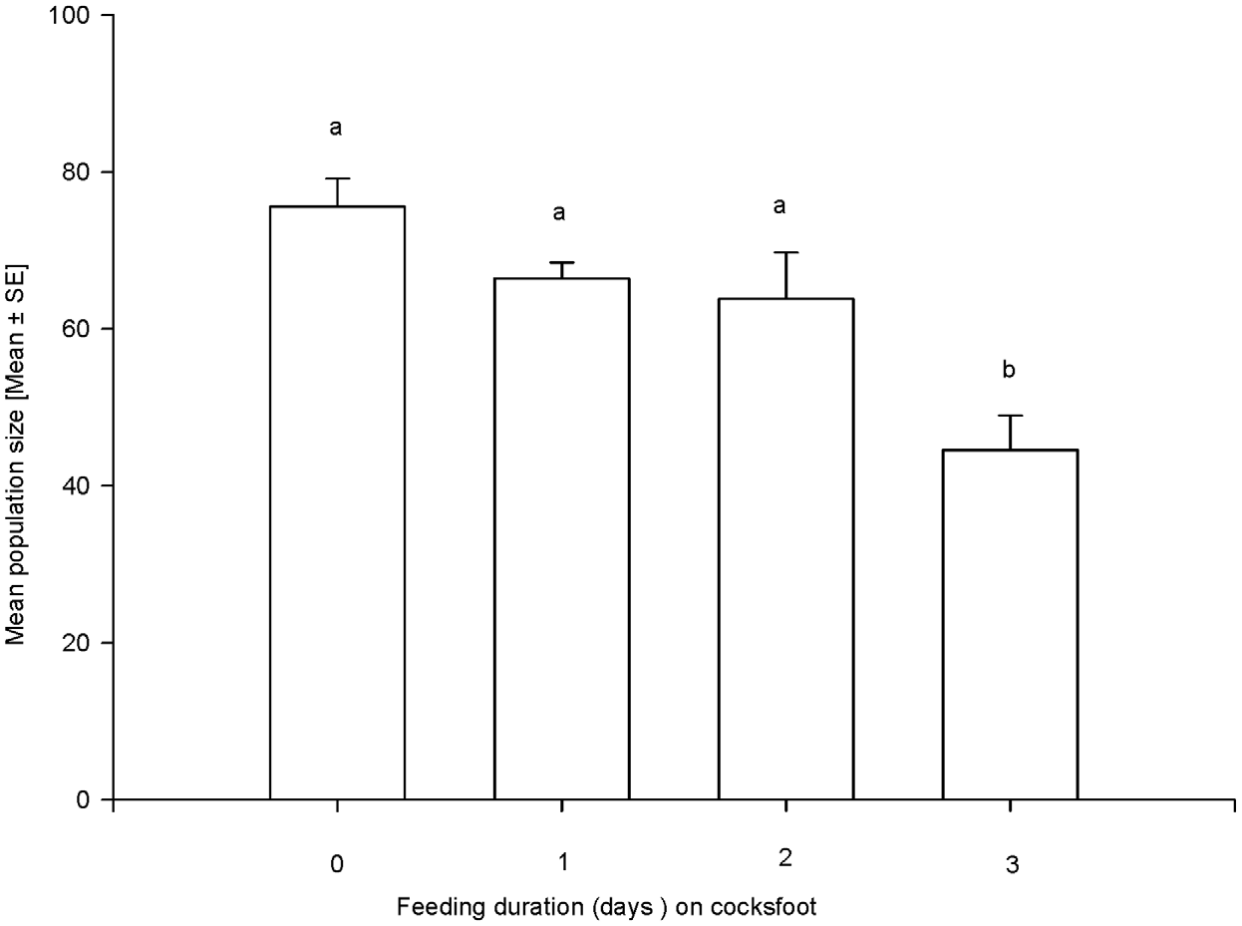
Effect of previous feeding duration on cocksfoot cultivar Amba on the performance of *S. avenae* clone 5 when transferred to wheat. Nymphs were allowed to feed on Amba for several days prior to transferring them to wheat (F_3,36_ = 9,91, P<0.001; R^2^ = 0.45).

**Figure 5:**
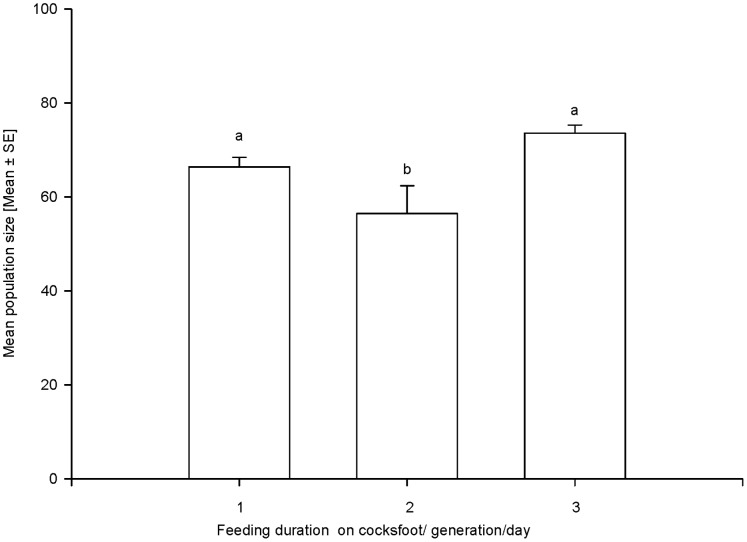
Effect of feeding duration on cocksfoot on the performance of *S. avenae* clone 5 on wheat; aphids fed many times with varying duration in different generations (F_2,27_ = 27.81, P<0.001; R^2^ = 0.67).

Feeding on cultivar Amba for more than 2 days caused a significant reduction in the number of offspring produced by *S. avenae* clone 5, when transferred back to wheat plants (F_3,36_ = 9.914, P<0.001; Figure 4). This effect was even evident for specimens of clone 5 which were allowed to adapt to Amba over the course of two generations (F_2,27_ = 27.810, P<0.001) (Figure 5). When the 3^rd^ generation of clone 5 was allowed to feed to Amba, its performance was enhanced as compared to the 2^nd^ generation; however, even these adapted specimens died when they were continuously caged on Amba for 1 month.

### Performance of Clone 5 on Cultivar Amba and on Different Host Plants When given a Choice

When nymphs of stage 1 and 2 were transferred to Amba, all of these nymphs died after 3 to 5 days, while none attained adult status. When stage 3 and 4 nymphs were used, most of them died in the first week, while only 2–5 out of 10 nymphs reached the adult stage. The few nymphs that reached the adult stage were winged and died after 2 to 3 days without producing any offspring.

Aphid speciemens were found to fed on different host plants, when given a choice between Amba, wheat, and two other grass species. Feeding continued for the first two weeks following transfer to these plants, but no surviving aphids were found after one month.

## Discussion

### Impact of Host Origin on Performance and Specialisation

The host plant species the clones have been collected from in the field, had no significant influence on clone performance. The results differ from those reported by [29], who observed the existence of specialized *S. avenae* clones on cocksfoot and concluded that a given clone performs better on the host plant of origin than on other host plants. Our results are also contradictory to the findings of [30], [31], and [32]. However, in these studies, the specialized genotypes of *S. avenae* on uncultivated host plants such as cocksfoot and oat were only determined using molecular markers, but not their clonal performance.

### Influence of WSC Levels on *S. avenae* Performance and Specialization on Cocksfoot

The results of our study showed that WSCs are correlated with the plant’s resistance to all tested clones of *S. avenae.* We were able to determine the threshold of WSC content in cocksfoot cultivars (>1.7% d.wt^-1^), responsible for an absolute resistance to *S. avenae* clones. Nutritional studies of aphids on artificial diets have already shown that they are not able to survive or reproduce on artificial diets containing a high content of sucrose (higher than 34% w/v) [13].

The osmotic pressure of the aphid’s haemolymph has been shown to increase with an increase in dietary sugar concentration, resulting in reduced performance and mortality due to osmotic stress [6]. Water-soluble carbohydrates in the phloem of cocksfoot consists mainly of sucrose, glucose, fructose and fructan [33]. The cocksfoot cultivars used in this study had a WSC content ranging from 1 to 8% d.wt^-1^; therefore, the mortality of all *S. avenae* clones on *D. glomerata* cultivars may be explained by the sugar barrier [6]. The correlation between the concentration of WSC in cocksfoot and the performance of *S. avenae* clones is very high. A decrease in the concentration of WSC was associated with increased aphid populations for all tested clones (Figure 2). *S. avenae* clones survived on cultivars which had less than 1.7% WSC (Table 2). Thus, the WSC concentration is correlated with the performance of *S. avenae* clones in cocksfoot. Furthermore, our results show that *S. avenae* clones cannot adapt to cocksfoot cultivars which have more than 1.7% of WSC, such as Amba. Thus, our results on cocksfoot differ from those of [29], who concluded that *S. avenae* clones adapt to their host plants and observed that performance of clones collected from wheat and transferred to cocksfoot hosts improved after three generations. Our results are in agreement with those of [29], who reported the specialization of *S. avenae* clones on the *Dactylis* cultivar Prairial (1.7% WSC).

**Table 2:** Origin, concentration of water soluble carbohydrates (WSCs), and survival of *Sitobion avenae* clones on Cocksfoot cultivars.

Cultivar	Origin	WSCconcentration [%]^1^	Survival
Glorus	Sweden	8.3	No
Dedinovskaya	Russia	7.8	No
Hayking II	Japan	6.1	No
Grassland'skara	New Zealand	3.4	No
Amba	Denmark	3.2	No
Milona	Czech Republic	2.2	No
Bartyle	Netherlands	2.1	No
Prairial	France	1.7	Yes
Barata	Netherlands	1.6	Yes
Dorise	Netherlands	1.1	Yes
Neva	Russia	1	Yes

1% dry weight.

Prior to the present study, no evidence had been published linking a plant’s WSC concentration with its resistance to *S. avenae*. Previously published research [34] was unable to determine which among the food coefficient, defined as ratios of the contents of soluble carbohydrates, total nitrogen, free amino acids, and other plant components, determines the resistance of wheat plants to *S. avenae*.

There have been no prior reports concerning the accumulation of chemical compounds in *Dactylis* cultivars during the selection for high WSC. An accumulation of phenols [35] and silicates [36] has been found in certain sub-species of *Dactylis*. However, *S. avenae* has been found to be unaffected by the presence of silicates [37]. *S. avenae* contains polyphenol oxidase and peroxidase enzymes in its saliva [38], which effectively decreases the phenol level of its host plant [39].

### Influence of Secondary Bacterial Endosymbionts on Tolerance of *S. avenae* Clones to WSC and Host Specialisation of *S. avenae*

In this study bacterial endosymbionts did not influence the performance of *S. avenae* clones on cocksfoot cultivars with WSCs more than 2.2%, which did not allow survival of all aphid clones. However, secondary bacterial endosymbionts effected the performance of aphid clones on cocksfoot cultivars with less than 2.2% WSC (F_1,187_ = 5.572, P<0.05). In this study, the clonal performance of *S. aveane* on cocksfot significantly colrelated with genotype of aphids (F_5,187_ = 62.940, P<0.001) and WSC (F_3,187_ = 444.657, P<0.001) and the we could did not assess the impact of the symbiotic bacteria because the lack of matrix. Only two genotypes (genotypes A and V) of those hrabouring the bacteria *Regiella insecticola* were tolerant to WSC and they could colonize the *D. glomerata* cultivar Prairial as compared to the other genotypes which harbouring the same or other bacteria. While only clone five form the clones which harboured *Hamiltonella defensa* was found to be partially tolerant to WSC but it was only able to survive for 1 month on *D. glomerata* cultivar Prairial. This finding, in addition to evidence that not all clones which harbour *Regiella insecticola* can survive on Prairial, indicates that the secondary bacterial endosymbionts are not responsible for specialization but their presence enhances clonal performance.

Our results differ from those reported by Douglas et al. [13], who found that the presence of *R. insecticola* negatively effected the clonal performance of pea aphids on artificial diets with sucrose concentration from 0.25 to 1 M. They also found that the presence of *H. defensa* had no impact on clonal performance. Moreover, they did not find positive or negative effects associated with the presence of bacterial endosymbionts on diets with sucrose concentrations higher than 1 M: they also failed to find any consistant pattern of performance in clones harbouring identical secondary bacterial endosymbionts.

There are many inferences drawn from the clonal performance of pea aphids concluding that the presence of secondary bacterial endosymbionts affects the performance of clones, but not their specialization. For example, when the performance of *R. insecticola*, *Serratia symbiotica* and *H. defensa*-harbouring clones of *A. pisum* was compared on clover as a host plant, it was found that the former outperformed the later by 100% [15]. On the other hand, when comparisons were made on alfalfa as a host plant, *H. defensa*-harbouring clones had a 50% better performance than *S. symbiotica* -harbouring clones, while *R. insecticola-* harbouring clones died on alfalfa [15]. Removing *R. insecticola* from these clones did not result in survival of *A. pisum* clones on alfalfa [40]. In Japan, pea aphid clones harbouring *R. insecticola* performed better on clover than on vetch [16]. However, up to now there is no conclusive evidence for the role of secondary bacterial endosymbionts on host specialization in *A. pisum* or its survival on artificial diets [6], [13], [40]. The authors of these studies speculate that the observed variability in their studies was due to a large genetic co-variability in both aphids and endosymbionts. However, when analysing the genetic variability in *R. insecticola* and *H. defensa* we found that the variability was limited, both within the same aphid species and across different aphid species (Alkhedir, personal observation). This finding further points to the importance of the interaction between host plants and clones in the aphid specialization process.

Our study demonstrates, for the first time, the role of WSC content in natural resistance of host plants to *S. avenae*. While the selection for high WSC might have a short-term positive impact on plant production, it may also impose long-term negative effects on the environment and biodiversity, for instance regarding the effect on other herbivorous pests, plant pathogens and their antagonists. Such cultivars should therefore be evaluated in a multitrophic context.

## Materials and Methods

### Host Plants Used in Experiments

Cocksfoot (*D. glomerata* L. cv. ‘Amba’) was purchased from C. Appel Company (Germany). All other cultivars were purchased from the National Agricultural Research Center for the Hokkaido Region - Japan and were analysed for WSC content according to Sanada et al. (2004); the results are shown in Table 2. All plants used in the study were grown in a greenhouse at 25±1°C, 50±5% humidity and a 16:8 hour light/dark regime at an illumination of 200 µE photon flux density. Seeds of the cultivars were separately germinated in trays and after one week, transplanted to small pots with a diameter of 11 cm. The growth medium in the pots was a 2∶1 mixture of soil (Fruhstorfer Typ P) and sand. Seedlings were used as plant hosts for aphids at 1 month of age. The plants were not fertilised during the study but they were regularly watered once each morning.

### Aphid Cultures and Rearing Procedures

In 2004 *Sitobion avenae* clones were collected from different regions (Goettingen, Kassel and Giessen) in central Germany by sweep-net sampling from wheat (*Triticum aestivum* L.), cocksfoot (*D. glomerata*) and graminoid grasses. 65 clones were established from single aphids kept on 7-day-old wheat seedlings (winter wheat cultivar ‘Bussard’; Lochow Petkus Company, Germany). These wheat seedlings had been grown in pots (11 cm diameter) filled with a 2∶1 mixture of soil (Fruhstorfer Typ P) and sand. The pots were covered with transparent ventilated cylindrical tubes measuring 10 cm×30 cm in size. Aphids were transferred to new plants every second week using a fine brush and the cultures were kept in a climate-controlled chamber (Viessmann Company, Germany) at a temperature of 20°C, 16:8 hour light/dark conditions, 60–80% humidity, and an illumination of 200 µE photon flux density. Wheat plants were not fertilized for the duration of the experiments but water was applied twice a week. Given these conditions, all clones reproduced parthenogenetically.

The 10 clones used in the study (Table 1) were selected based on five microsatellite loci (see Protocol S1 and Table S1) representing 6 common genotypes, 4 of which regularly occur in central Germany. Nine of the clones harbour secondary bacterial endosymbionts (i.e. candidatus *Regiella insecticola* or candidatus *Hamiltonella defensa* (Protocol S1 and Table 1).

### Performance of Selected Clones on Cocksfoot Cultivars

In these experiments we tested whether the common clones of *S. avenae* are specialized on cocksfoot aiming at understanding the most important parameters shaping the relationship between *S. avenae* clones and cocksfoot. Moreover, we tested whether the host plant origin of the clones or the collecting sites contributed to their specialization on cocksfoot.

The performance of *S. avenae* selected clones (Table 1) was tested on ten *D. glomerata* cultivars (Table 2) by determining the population growth after one month following the transfer of 10 synchronized first stage nymphs on each of these cultivars. This experiment was done with five replications using the standard rearing conditions described above. Population size was monitored weekly. The surviving specimens on cultivar Prairial were reared on wheat plants for 2 weeks and subsequently transferred to Prairial for 1 month.

### Performance of Different Clones on Cultivar Amba

The cocksfoot cultivar Amba was used as a standard for the experiments, because it is commercially used in Germany as a forage grass. In these experiments we tested if Amba can be colonized by different *S. avenae* clones. Seedlings of Amba were grown in a greenhouse, as described above, and then transferred to a growth chamber. All 65 collected clones (Table S1) of *S. avenae* were reared on Amba with five synchronized fourth-stage nymphs as a starter population for one month under the rearing conditions described above.

### Performance of Clone 5 on Wheat Following Feeding on Amba

The effect of duration of prior feeding on cocksfoot cultivar Amba on the performance of *S. avenae* clone 5 on 7 day-old wheat seedlings was measured two weeks after transfering clone 5 nymphs from Amba. We used clone 5 for this experiment, because in our previous experiments, and contrary to the other clones tested, many winged specimens were produced. Prior to propagating the nymphs of clone 5 on wheat, they were fed on Amba using two different experimental procedures. In the first procedure, nymphs were caged for 1, 2, or 3 days on Amba leaves using clip-cages with a diameter of 9 cm; then five synchronized fourth stage nymphs were transferred to wheat plants and caged on the leaves for two weeks. In the second procedure, nymphs that had previously been caged on Amba for one day (see above), were caged on Amba for 2 days after which five synchronized fourth stage nymphs were transferred and caged on wheat leaves for two weeks. Nymphs (previously caged on Amba for 2 days in the second procedure) were caged on Amba for 3 days after which five synchronized fourth stage nymphs were transferred and caged on wheat leaves for two weeks. This experiment was performed with 10 replications for each feeding treatment. The set-up of this experiment is explained in Figure S1. All plants with aphids were incubated in the same growth chamber in which all previously described experiments were performed and handled as described in the previous experiments.

### Supplementary Experimentes

These experiments aimed at understanding whether survival of clone 5 nymphs on Amba differs between aphid growth stages. Moreover, we tested wether specimens of clone 5 will avoid Amba given a choice with other host plants and whether feeding on Amba will result in reduced performance, even after transferred to other host plants. In order to assess the performance of different growth stage of clone 5 on Amba, we incubated 5 nymphs from each growth stage separately on Amba seedlings under experimental conditions identical to those described above. To test the clonal performance on a mix of different host plants, we grew four different plant species in one pot, i.e. *D. glomerata* “Amba”, wheat (cultivar Bussard), bluegrass (*Poa annua* L., unknown cultivar), and ryegrass (*Lolium perenne* L. cv. ‘Herault’). 10 synchronized first stage nymphs were transferred to these pots, when plants were three weeks old. Seeds of bluegrass and ryegrass were obtained from Appels Wilde Samen Company, Germany. This experiment was performed at the same conditions as the other experiments with 10 replications; populations size was monitored weekly and finally assessed after one month.

### Statistical Analysis

General linear models (GLM) were used to analyse the relationship between WSC levels, presence of secondary endosymbionts, host plant origin of clone, collecting site and genotype on clonal performance. Mean population size at the time the experiments were terminated was used as the dependent factor, while all other factors were treated as independent categorical variables. Zero values were excluded from the analyses; due to a lack of a matrix we were not able to nest the categorical variables. ANOVA was also used to analyse the effect of WSC concentrations and secondary endosymbionts on clonal performance. Population size was used as the dependent factor while WSC concentrations, bacterial endosymbionts and their interaction were treated as independent factors. Fisher's least significant difference (LSD) adjustment was used to compare clonal performance. ANOVA was used for analysing the effect of feeding duration on cocksfoot Amba on clonal performance, using the mean population size as the dependent factor and feeding duration as the independent factor. In the analysis of the performance of *S. avenae* clones on wheat after feeding on Amba, mean population size was used as the dependent factor and feeding duration on Amba as the independent factor. Fisher's LSD adjustment was used to analyse the effect of feeding duration. Systat for Windows version 12.01.02 [41] was used to perform these analyses.

## Supporting Information

Figure S1 **Experimental scheme for testing the influence of duration of prior feeding on cocksfoot cultivar Amba on the performance of** ***S. avenae*** **clone 5 nymphs on wheat seedlings measured two weeks after transfering them from Amba.**
(TIFF)

Table S1 **Genotype profiles of** ***S. avenae*** **clones used in the study.**
(DOCX)

Protocols S1 **DNA extraction, microsatellite genotyping, and symbiotic bacteria identification protocols for** ***Sitobion avenae*** **clones used in the study.**
(DOCX)
