# Supplementary figures and images for: Relationship between Water Soluble Carbohydrate Content, Aphid Endosymbionts and Clonal Performance of Sitobion avenae on Cocksfoot Cultivars
Source: PLoS One. 2013 Jan 14;8(1):e54327. doi: 10.1371/journal.pone.0054327 (PMC3544763; doi:10.1371/journal.pone.0054327)

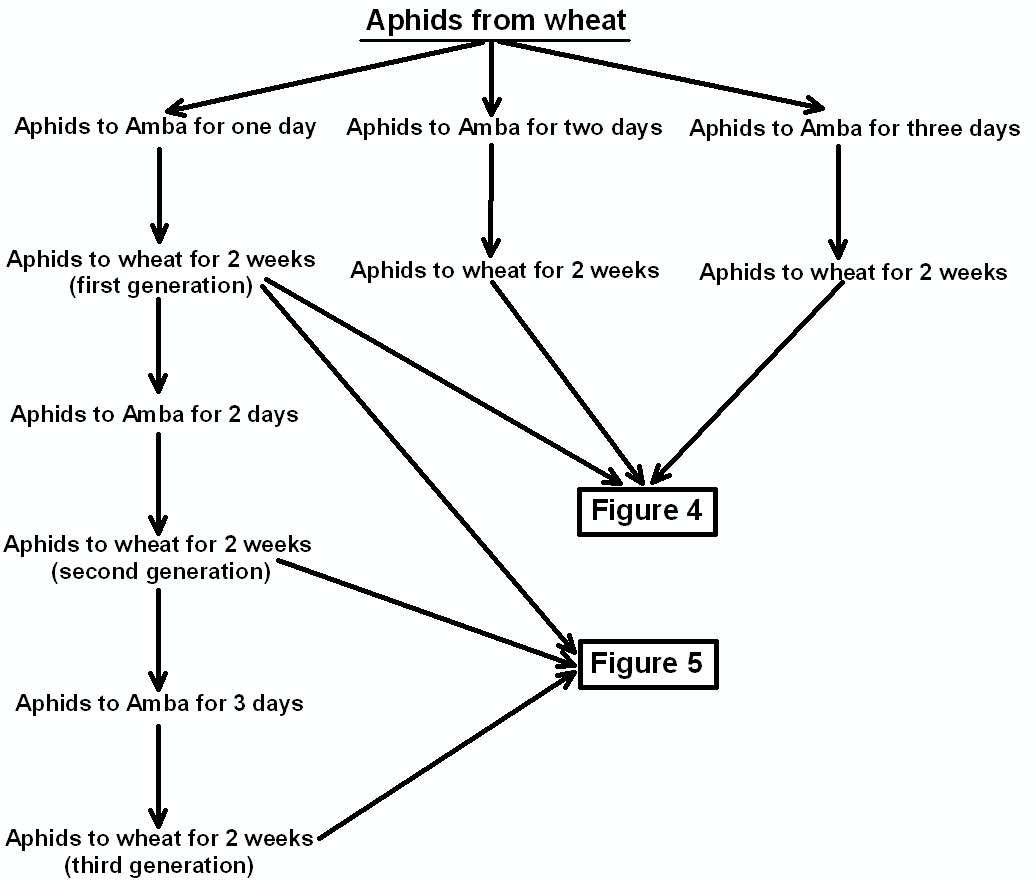

Supplement: Figure S1 — Experimental scheme for testing the influence of duration of prior feeding on cocksfoot cultivar Amba on the performance of S. avenae clone 5 nymphs on wheat seedlings measured two weeks after transfering them from Amba. (TIFF) [file pone.0054327.s001.tiff]
