# Supplementary material for: Relationship between Water Soluble Carbohydrate Content, Aphid Endosymbionts and Clonal Performance of Sitobion avenae on Cocksfoot Cultivars
Source: PLoS One. 2013 Jan 14;8(1):e54327. doi: 10.1371/journal.pone.0054327 (PMC3544763; doi:10.1371/journal.pone.0054327)
Supplement: Table S1 — Genotype profiles of S. avenae clones used in the study. (DOCX) [file pone.0054327.s002.docx]

**Table S1.** Genotype profiles of *S. avenae* clones used in the study.

| Clone^2^ Locus | Sm10^1^ | Sm11 | Sm17 | S16b | Sa4Σ | Number of individuals collected  2001–2003 |
| --- | --- | --- | --- | --- | --- | --- |
| 1-2-3-19-20 | 164/166 | 144/149 | 178/179 | 209/274 | 168/168 | 194 |
| 7-11-13-14 | 152/166 | 144/144 | 178/178 | 173/189 | 156/172 | 115 |
| 4-5-6-29-30-31-32-33-34-35-36 | 164/166 | 144/144 | 179/179 | 159/173 | 162/168 | 66 |
| 8 | 164/166 | 144/148 | 178/178 | 173/173 | 156/162 | 3 |
| 9 | 164/164 | 144/148 | 178/178 | 179/275 | 162/172 | 0 |
| 10 | 160/164 | 144/149 | 178/178 | - | 162/168 | 0 |

^1^Units (bp).

^2^clones which were collected only once are not listed, except for clones 9 and 10, selected for further experiments
