# Supplementary material for: Relationship between Water Soluble Carbohydrate Content, Aphid Endosymbionts and Clonal Performance of Sitobion avenae on Cocksfoot Cultivars
Source: PLoS One. 2013 Jan 14;8(1):e54327. doi: 10.1371/journal.pone.0054327 (PMC3544763; doi:10.1371/journal.pone.0054327)
Supplement: Protocols S1 — DNA extraction, microsatellite genotyping, and symbiotic bacteria identification protocols for Sitobion avenae clones used in the study. (DOCX) [file pone.0054327.s003.docx]

**Protocol S1.** DNA extraction, microsatellite genotyping, and symbiotic bacteria identification protocols for *Sitobion avenae* clones used in the study.

**DNA extraction**

Aphids and parasitoids were collected and stored at -20°C until the DNA extraction. DNA were extracted from all insects from this study and from aphids which were screened for the endosymbionts using protocol published by Sunnucks et al [[1](#_ENREF_1)]. The quality of extracted DNA was checked on 0.8% agarose gels and visualized by staining with ethidium bromide.

**Microsatellite genotyping**

DNA was extracted from individual aphids following the ‘salting out’ protocol of [[1](#_ENREF_1)]. Genotypes were determined at five microsatellite loci: Sm10, Sm11, Sm17 [[1](#_ENREF_1)], Sa4∑ [[2](#_ENREF_2)] and S16b [[3](#_ENREF_3)]. Sm11 is linked to the X-chromosome [[4](#_ENREF_4)], whereas all the other loci are autosomal [[2](#_ENREF_2)]. Polymerase chain reactions (PCRs) were performed in 10 µl volumes containing 0.5 units of recombinant *Taq* (rTaq TAKARA BIO INC, Japan) polymerase, Mg^2+^-free reaction buffer, 200 µM dNTPs (TAKARA BIO INC, Japan), 5 pmol of each primer (ROTH), 10% DMSO, 2 mM Mg^2+^ (as MgCl_2_) and 2 µl of 10-fold diluted DNA extract (approx. 2–10 ng) (for the Sm17 locus) or 2.5 mM Mg^2+^ and 5.8 µl of DNA extract (5.8–29 ng) (for all other loci). All PCRs were performed in a BIOMETRA gradient thermocycler with initial denaturation at 94°C for 2 min. This was followed by five touch-down cycles (first cycle 7°C higher than the final annealing temperature), 29 cycles of denaturation at 94°C for 15 s, and annealing of primers for 30 s at 55°C (Sm17 and Sm10 loci), 47°C (Sm11 locus) and 60°C (S16b and Sa4∑). Elongation took place at 72°C, ending with a 10-min termination step at the same temperature. Gel electrophoresis in 6% denaturing polyacrylamide gel and silver staining were performed as described by [[5](#_ENREF_5)]. Allele sizes were estimated using a sequencing size ladder prepared by sequencing the pGEM-3Zf(+) vector (PROMEGA) using the Thermo Sequenase Cycle Sequencing Kit (USB, USA). Gels were documented using a digital camera. Image analysis was performed with the Digitrace software package (IMATEC Elektronische Bildanalysesysteme GmbH, Germany).

**Identification of symbiotic bacteria**

Secondary endosymbionts extracted from aphid clones, were identified using DNA extracted from specimens either by end-point PCR or by real-time PCR and confirmed by sequencing PCR products. Specific primers described by Douglas et al [[6](#_ENREF_6)] for *Regiella insecticola, Hamiltonella defensa* or *Serratia symbiotica* were used to amplify the 16S ribosomal RNA gene partially or completely. Amplification and melting curve analyses were performed using an iCycler (BioRad, Hercules, CA, USA). The reaction mixture consisted of 1X NH4 reaction buffer (Bioline, Luckenwalde, Germany), 1.8 mM MgCl2 (Bioline, Luckenwalde, Germany), 0.2 mM of each dATP, dTTP, dCTP and dGTP (Bioline, City, Germany), 0.5 µM of each primer, 0.4 u BIOTaq DNA polymerase (Bioline, Luckenwalde, Germany), 10 nM fluorescein (BioRad, Hercules, CA, USA), 100,000-times diluted SYBR Green I solution (Invitrogen, Karlsruhe, Germany), 6 µl of template DNA and ddH2O up to 24 µl.

**References**

1. Sunnucks P, England PR, Taylor AC, Hales DF (1996) Microsatellite and chromosome evolution of parthenogenetic Sitobion aphids in Australia. Genetics 144: 747-756.

2. Simon JC, Baumann S, Sunnucks P, Hebert PDN, Pierre JS, et al. (1999) Reproductive mode and population genetic structure of the cereal aphid Sitobion avenae studied using phenotypic and microsatellite markers. Molecular Ecology 8: 531-545.

3. Wilson ACC, Massonnet B, Simon J-C, Prunier-Leterme N, Dolatti L, et al. (2004) Cross-species amplification of microsatellite loci in aphids: assessment and application. Molecular Ecology Notes 4: 104-109.

4. Wilson ACC, Sunnucks P, Hales DF (1997) Random loss of X chromosome at male determination in an aphid, Sitobion near fragariae, detected using an X-linked polymorphic microsatellite marker. Genetical Research 69: 233-236.

5. Llewellyn KS, Loxdale HD, Harrington R, Brookes CP, Clark SJ, et al. (2003) Migration and genetic structure of the grain aphid (Sitobion avenae) in Britain relates to climate and clonal fluctuation as revealed using microsatellites. Molecular Ecology 12: 21-34.

6. Douglas AE, Francois CLMJ, Minto LB (2006) Facultative 'secondary' bacterial symbionts and the nutrition of the pea aphid, Acyrthosiphon pisum. Physiological Entomology 31: 262-269.
